# Intra- and post-operative risk of retinal breaks during vitrectomy for macular hole and vitreomacular traction

**DOI:** 10.1371/journal.pone.0272333

**Published:** 2022-08-11

**Authors:** Neil K. Jairath, Yannis M. Paulus, Angela Yim, Yunshu Zhou, Bela J. Parekh, Ruple Jairath, David C. Musch, Julie M. Rosenthal

**Affiliations:** 1 Department of Ophthalmology and Visual Sciences, University of Michigan, Ann Arbor, MI, United States of America; 2 Department of Biomedical Engineering, University of Michigan, Ann Arbor, MI, United States of America; 3 Department of Epidemiology, University of Michigan, Ann Arbor, MI, United States of America; Massachusetts Eye & Ear Infirmary, Harvard Medical School, UNITED STATES

## Abstract

**Background/Objective:**

To evaluate the development of intra- and post-operative retinal breaks after pars plana vitrectomy (PPV) for macular hole (MH) and/or vitreomacular traction (VMT).

**Subjects/Methods:**

Medical records of patients who underwent PPV at Kellogg Eye Center between 1/1/2005–6/30/2018, were evaluated in three groups: group 1, MH/VMT (n = 136); group 2, epiretinal membrane (ERM) without VMT (n = 270); and group 3, diagnostic vitrectomy (DV) or vitreous opacities (n = 35). Statistical analyses were conducted using SAS.

**Results:**

20.6% of patients with MH/VMT, 8.5% of patients with ERM, and 5.7% of patients with DV or vitreous opacities had either intra-operative or post-operative breaks. Indication of MH/VMT versus ERM was a significant predictor for this outcome (p = .0112). The incidence of retinal breaks was higher in operations using 23-gauge versus 25-gauge PPV (25.0% vs. 7.4%, p < .0001).

**Conclusions:**

The presence of MH and/or VMT is a significant risk factor for retinal breaks from PPV, as is use of 23-gauge vitrectomy.

## Introduction

While pars plana vitrectomy (PPV) has transformed access to the posterior segment to treat a variety of vitreoretinal diseases and restore patients’ vision and quality of life [1], as with any procedure, it carries a risk of complications, including elevated intraocular pressure, corneal epithelial defects, endophthalmitis, and peri-operative retinal breaks. Although the incidence of serious complications is low [2, 3], given their morbidity, it is essential that physicians have information regarding the risks, benefits, and expected post-operative course to counsel and manage patients appropriately. Indications for PPV include visually significant epiretinal membrane (ERM), vitreomacular traction (VMT), rhegmatogenous and tractional retinal detachments, macular hole (MH), vitreous hemorrhage, and intraocular tumors, among many others. Although the indications for vitrectomy are numerous, there are limited data on the relative rate of complications for each.

Intra-operative retinal breaks or tears are well-known complications of vitrectomy that may lead to morbidity, vision loss, and/or re-operation. Rates of retinal breaks after PPV have been reported to range from 1.8% to 36.9% [3–8]. One retrospective study evaluated 79 patients with MH and 41 patients with ERM for the development of intra-operative retinal breaks, reporting increased incidence of retinal breaks in patients with MH (35.4% vs. 4.9%, p = 0.0006) [9]. Another study evaluated the incidence of iatrogenic retinal breaks in 176 patients undergoing 20- and 23-gauge vitrectomy, finding that an indication of MH carried a nine-fold greater risk of iatrogenic retinal breaks than an indication of ERM (11.7% vs. 1.3%) [10]. Studies evaluating the risk of retinal detachment after vitrectomy specifically for ERM, MH, and VMT found it to be 1.1% [11]. Given these data, some groups have proposed prophylactic laser at the sclerotomy sites to reduce the risk of post-operative retinal breaks [12–14].

VMT involves an abnormally raised foveal contour with focal attachment of the vitreous cortex, which may lead to decreased visual acuity attributed to anteroposterior or axial traction on the macula [15, 16]. Recent literature suggests that vitreous traction can lead to MH development [17]. MH is defined by the presence of a retinal defect in the center of the fovea, causing central vision impairment [18]. In the general population, the prevalence of MH is reported to be around 3.3 per 1,000 people [19]. This study was designed to identify whether these patients may be at higher risk for intra- and post-operative retinal breaks, as they may have certain characteristics, such as altered vitreous and vitreoretinal adhesion, that predispose them to the development of intra-operative breaks or adhesion during surgery.

## Materials and methods

### Subjects and data collection

This is a retrospective, nonrandomized comparative case series. Medical records of all consecutive patients who underwent PPV at the Department of Ophthalmology and Visual Sciences at the University of Michigan Kellogg Eye Center between January 1, 2005, and June 31, 2018, were collected to identify those patients who underwent PPV for MH/VMT, vitreous opacities, ERM without VMT, or diagnostic purposes. All patients underwent 23-, 25-, or 27-gauge vitrectomy. Data was accessed between September 1, 2019 and April 6, 2021. Inclusion criteria included the diagnoses listed above. Exclusion criteria included prior PPV, vitreous hemorrhage, panretinal photocoagulation, proliferative diabetic retinopathy, focal laser, history of retinopathy of prematurity, any diagnoses affecting the vitreoretinal interface, and/or age<40 years. The primary outcome was the incidence of combined post-operative and intra-operative retinal breaks. Secondary outcomes included incidence of post-operative retinal breaks alone, relation of pre-operative breaks to incidence of intra- and post-operative breaks, and relation of the primary outcome to post-operative visual acuity (VA) and phakic status.

The following data were collected from the patient health record and operative report: age, sex, procedure date, PPV indication, presence of myopia (defined as spherical equivalent less than 0 diopters), procedure details including laterality, gauge, treatment provided, use of perfluoropropane (C3F8) or sulfurhexafluoride (SF6) gas, ERM peel, internal limiting membrane (ILM) peel, endolaser, indocyanine green, triamcinolone or Triesence, presence, number, and location of intra-operative breaks, peri-operative complications, VA preoperatively and longitudinally up to 6 months after the procedure, fundus exams preoperatively and longitudinally up to 6 months after the procedure, pre-operative breaks, post-operative breaks, optical coherence tomography testing, and associated medical history.

This study protocol was approved by the Institutional Review Board for Human Subjects Research at the University of Michigan (HUM00122296) and adhered to the tenets of the Declaration of Helsinki.

### Data analysis

The differences between patient groups were analyzed using the Mann-Whitney U test, chi-square or Fisher exact tests. Continuous variables are described by means (with standard deviations) and categorical variables are described by frequencies and percentages [N (%)]. Phakic status, gauge, and indication for vitrectomy were analyzed as explanatory variables. Univariate analysis and multivariable logistic regression were conducted to evaluate the association of these factors with intra-operative breaks, post-operative breaks, and combined intra-operative and post-operative breaks (MH/VMT as reference group) using indication, age, gender, phakic status, history of diabetes mellitus, presence of myopia, and PPV gauge (25-gauge as reference group) as explanatory variables. Statistical analysis was conducted in SAS version 9.4 (SAS Institute, Cary, NC, USA).

## Results

### Baseline and treatment characteristics

**Fig 1:**
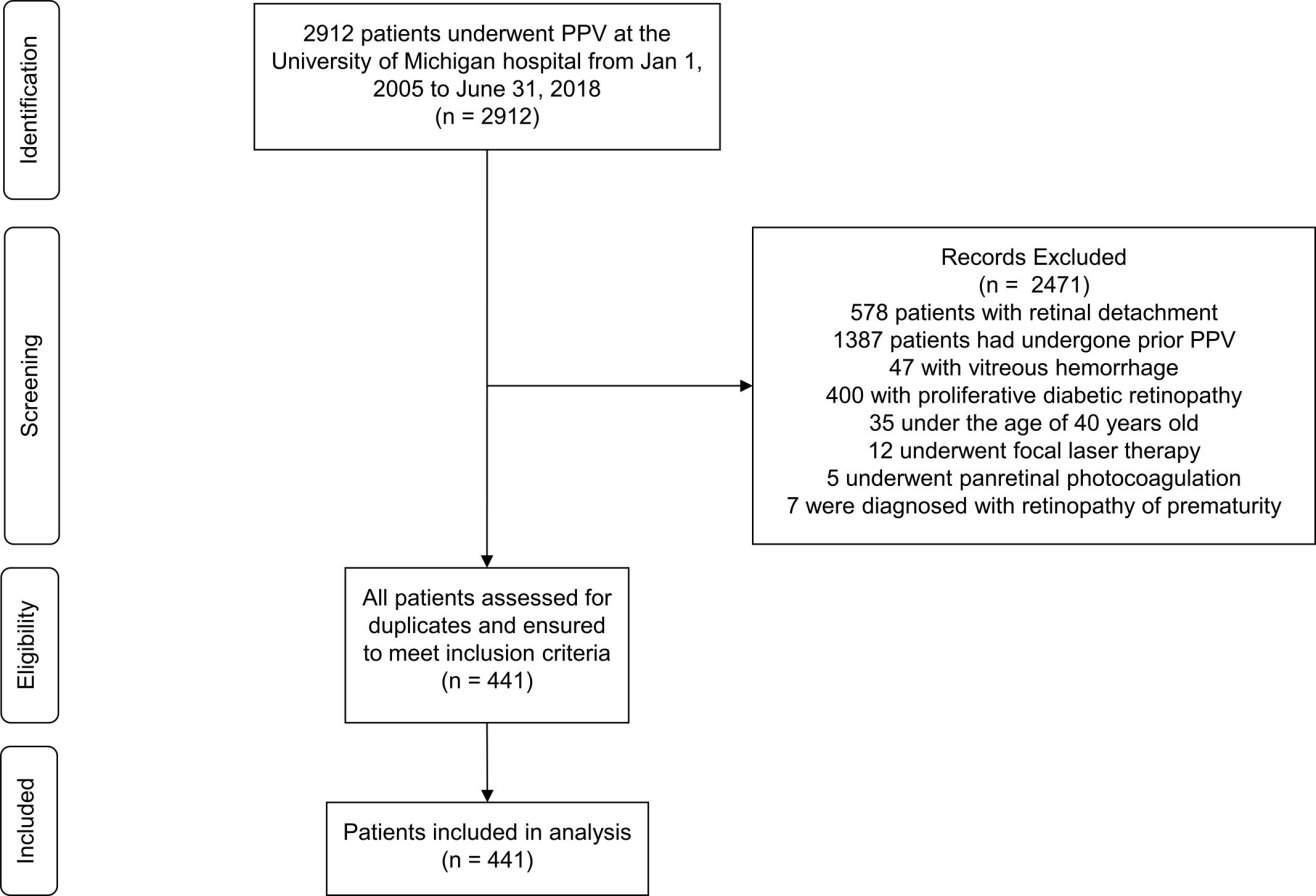
Flow diagram detailing exclusion criteria for the patients acquired in the study, including numbers of patient records removed at each exclusion step that led to curation of the final group of patient records included in the analysis. Of 2,912 patient records retrieved with the initial search strategy, 441 were included in the study.

**Fig 1** displays the search and exclusion criteria applied in the study. After applying exclusion criteria, 441 patients were included in the analysis. These patients were divided into three groups determined by the hypothesized level of risk for intra-operative retinal breaks (highest to lowest): group 1 (n = 136) included patients with MH and VMT, group 2 (n = 270) contained patients with ERM, and group 3 (n = 35) included patients who underwent diagnostic vitrectomy (DV) or had vitreous opacities.

Baseline patient factors and primary outcome information divided by group can be found in **Table 1**. Additionally, the mean refractive status of the patients studied was -0.7 diopters (D), ranging from -10.0 D to +9.8 D, with standard deviation (SD) of 3.1 D. Average preoperative vision, as measured by logarithm of the minimum angle of resolution (logMAR) was 0.6, or Snellen equivalent 20/80 (SD = 0.4), improving to 0.4, or Snellen equivalent 20/50 (SD = 0.3) at 3–6 months follow-up. 24 patients were lost to follow-up before the 3-month post-operative checkup. Of the patient procedures recorded, 151 (34.2%) used C3F8 gas, 9 (2.0%) used SF6 gas, 139 (31.5%) used endolaser or cryotherapy, 153 (34.7%) used indocyanine green, 213 (48.3%) used triamcinolone, 284 (64.4%) included ILM peel, and 89 (20.2%) included ERM peel. A full summary of patient treatment characteristics may be found in **Table 2.**

**Table 1:** Patient demographic data and primary outcome measures by group.

Baseline Factors and Primary Outcomes	MH/VMT (N = 136)	ERM (N = 270)	DV/VO (N = 35)	*p-*value
Mean Age at Surgery in Years (SD)	73.2 (11.9)	72.7 (11.4)	71.6 (10.7)	0.6500
Gender				0.5732
Male	76 (55.9%)	137 (50.7%)	20 (57.1%)	
Female	60 (44.1%)	133 (49.3%)	15 (42.9%)	
Laterality				0.1992
Left Eye	64 (47.1%)	127 (47.0%)	22 (62.9%)	
Right Eye	72 (52.9%)	143 (53.0%)	13 (37.1%)	
Gauge				0.0003*
23	51 (37.5%)	65 (24.1%)	8 (22.9%)	
25	80 (58.8%)	205 (75.9%)	27 (77.1%)	
27	5 (3.7%)	0	0	
Myopia	78 (57.8%)	172 (64.2%)	22 (62.9%)	0.4556
Lens Status				0.0031*
Cataract (Phakic)	94 (69.1%)	214 (79.3%)	33 (94.3%)	
Pseudophakic	42 (30.9%)	56 (20.7%)	2 (5.7%)	
Comorbidities				
Hypertension	32 (23.5%)	57 (21.1%)	11 (31.4%)	0.3748
Diabetes Mellitus	26 (19.1%)	44 (16.3%)	2 (5.7%)	0.1603
Median Follow-Up (Days)	629.5	659.0	661.0	0.1951
Pre-operative Retinal Break	8 (5.9%)	9 (3.3%)	1 (2.9%)	0.4390
Intra-operative Retinal Break	22 (16.2%)	12 (4.4%)	2 (5.7%)	0.0002*
Post-operative Retinal Break	8 (5.9%)	12 (4.4%)	0	0.3266
Intra- or Post-operative Break	28 (20.6%)	23 (8.5%)	2 (5.7%)	0.0010*

* Denotes Significance, *p* < 0.05

Abbreviations: SD, standard deviation; MH, macular hole; VMT, vitreomacular traction; ERM, epiretinal membrane; DV, diagnostic vitrectomy; VO, vitreous opacities. The first group in each category is designated as the reference group for statistical analyses

**Table 2:** Patient treatment characteristics by group.

Treatment Modality	MH/VMT (N = 136)	ERM (N = 270)	DV/VO (N = 35)
C_3_F_8_ gas	111 (81.6%)	20 (7.4%)	20 (57.1%)
SF_6_ gas	8 (5.9%)	1 (0.4%)	0 (0%)
Endolaser or Cryotherapy	75 (55.2%)	37 (13.7%)	27 (77.1%)
ICG	35 (25.7%)	117 (43.3%)	1 (2.9%)
Triamcinolone	66 (48.5%)	126 (46.7%)	21 (60.0%)
Epiretinal membrane peel	13 (9.6%)	75 (27.8%)	1 (2.9%)
Internal limiting membrane peel	73 (53.7%)	209 (77.4%)	2 (5.7%)

Rows indicate specific treatment modalities used in the PPV. Abbreviations: MH, macular hole; VMT, vitreomacular traction; ERM, epiretinal membrane; DV, diagnostic vitrectomy; VO, vitreous opacities; C3F8, perfluoropropane; SF6, sulfurhexafluoride; ICG, indocyanine green.

### Intra-operative and post-operative breaks

There were 22 (16.2%) patients with MH/VMT, 12 (4.4%) patients with ERM, and 2 (5.7%) patients with DV or vitreous opacities who developed an intra-operative retinal tear (p = 0.0002). Twenty-eight (20.6%) patients with MH/VMT, 23 (8.5%) patients with ERM, and 2 (5.7%) patients with DV or vitreous opacities had either intra-operative or post-operative breaks (p = 0.0010). There were a significantly higher number of patients with pseudophakia in the MH/VMT group (30.9%) than in both the ERM group (20.7%) and DV or vitreous opacities group (5.7%, p = 0.0031). **Table 1** displays complete primary outcome data. The multivariable logistic regression identified MH/VMT as being significantly predictive for the outcome of intra- or post-operative tear when compared to ERM (OR 0.618, 95% CI 0.205–1.858, p = 0.0112), including pre-operative breaks. Increasing age was also predictive for increased risk of intra-operative breaks (OR 1.059 per 1-year increase, 95% CI 1.024–1.095, p = 0.0001). Myopia, treated as a binary variable indicating presence or absence, was protective against intra- or post-operative breaks (OR 0.377, 95% CI 0.199–0.713, p = 0.0027). Complete multivariable analysis results are found in **Table 3**.

**Table 3:** Univariate and multivariable logistic regression analysis.

Predictors of Intraoperative or Postoperative Retinal Breaks				
Predictor	Univariate Odds Ratio (95% CI)	Univariate *p-*value	Multivariable Odds Ratio (95% CI)	Multivariable *p*-Value
MH/VMT vs. ERM	2.786 (1.533–5.051)	0.0008*	2.336 (1.214–4.504)	0.0112*
Age	1.060 (1.028–1.093)	0.0002*	1.059 (1.024–1.095)	0.0007*
Gender: F vs. M	1.188 (0.669–2.109)	0.5573	1.320 (0.698–2.495)	0.3936
Pseudophakia: Yes vs. No	1.150 (1.050–1.260)	0.8907	0.842 (0.395–1.796)	0.6563
DM: Yes vs. No	1.055 (0.490–2.270)	0.8907	0.785 (0.340–1.814)	0.5718
Myopia: Yes vs. No	0.383 (0.213–0.688)	0.0026*	0.377 (0.199–0.713)	0.0027*
Gauge: 23 vs. 25	4.394 (2.425–7.960)	<0.0001*	4.516 (2.369–8.609)	< .0001*

* Denotes significance, *p* < 0.05

Univariate and multivariable logistic regression analysis including odds ratio, 95% confidence interval, and *p-*value for individual comparisons (asterisks indicate p-values <0.05). The multivariable model evaluated each potential predictor upon adjusting for indication for vitrectomy, age of patient at time of surgery, gender, pseudophakia, diabetes mellitus, presence of myopia, and gauge of vitrectomy. Abbreviations: MH, macular hole; VMT, vitreomacular traction; ERM, epiretinal membrane; DV, diagnostic vitrectomy; VO, vitreous opacities; CI, confidence interval; F, female; M, male; DM, Diabetes Mellitus.

### Gauge of vitrectomy

The incidence of intra-operative breaks differed significantly between the 23-gauge and 25-gauge groups (19.4% vs. 3.8% respectively, p<0.0001). The incidence of intra- or post-operative breaks was also higher in operations using 23-gauge vitrectomies versus 25-gauge vitrectomies (25% vs. 7.4% respectively, p<0.0001). On multivariable logistic regression analysis, use of 23- vs. 25-gauge PPV was an independent predictor of the primary outcome measure (OR 4.516, 95% CI 2.369–8.609, p<0.0001). There was no difference in pre-operative breaks (p = 0.1225) or post-operative breaks alone (p = 0.3112) between the gauge groups. Notably, 23-gauge vitrectomy use was significantly higher in patients with MH/VMT than ERM and DV/vitreous opacities (37.5% versus 24.1% and 22.9%, p = 0.0003).

### Other secondary outcomes

Additional secondary outcomes included incidence of post-operative retinal breaks alone (**Table 1**), and relationship of the primary outcome to post-operative VA and phakic status. There was no significant difference in incidence of pre-operative breaks between the three study groups (p = 0.4390). The same was true for post-operative retinal breaks alone (p = 0.3266). There was no significant difference in the post-operative VA of patients with intra- or post-operative breaks at postoperative day one (p = 0.9956), post-operative week one (p = 0.6546), post-operative month one (p = 0.2534), or 3–6 months post-operative (p = 0.7919) compared to those patients without breaks (**Table 4**). There was no difference between patients who had undergone cataract surgery in the past, and those who had not (p = 0.7959). 18.9% (10 of 53) of patients with a retinal break progressed to retinal detachment. 81.1% (43 of 53) of patients with a retinal break underwent reoperation during the follow-up period of our study, including 100% of those with retinal detachment and 12.1% (47 of 388) of patients without retinal break underwent reoperation during the follow-up period of our study.

**Table 4:** Mean logMAR visual acuity by time after pars plana vitrectomy.

Mean Visual Acuity, by Time after PPV	Intra- or Post-operative Tear	Snellen Conversion	No Tear	Snellen Conversion	*p*-value
Mean VA one day post-operatively (SD) (n = 442)	2.3 (0.9)	20/3990	2.2 (1.0)	20/3170	0.9956
Mean VA one week post-operatively (SD) (n = 442)	1.0 (1.0)	20/200	1.1 (1.1)	20/250	0.6546
Mean VA one month post-operatively (SD) (n = 442)	0.4 (0.2)	20/50	0.4 (0.2)	20/50	0.2534
Mean VA three to six months post-operatively (SD) (n = 418)	0.3 (0.2)	20/40	0.4 (0.3)	20/50	0.7919

Visual acuity is expressed as logarithm of the minimum angle of resolution (logMAR) values with Snellen conversions. Abbreviations: PPV, pars plana virectomy; VA, visual acuity; SD, standard deviation.

## Discussion

This study sought to evaluate the incidence of intra-operative and post-operative retinal breaks in patients undergoing PPV for MH or VMT, compared to that of those undergoing PPV for vitreous opacities, ERM without VMT, or diagnostic vitrectomies, as well as to evaluate potential demographic, medical, or surgical risk factors for this complication. We found that the diagnosis of MH or VMT significantly increases the risk of intra-operative retinal breaks, in agreement with the hypothesis guiding this study. Additionally, 23-gauge vitrectomy appeared to confer greater risk of breaks than did 25-gauge vitrectomy.

Despite the continued evolution of vitreoretinal surgical techniques, the incidence of retinal breaks is still clinically significant. In small-incision vitrectomy, it has been reported to range from 0% to 36.9% of eyes [6–8, 10, 12, 20–22]. This variation may be explained by differences in indications for PPV, techniques used during surgery, or methods of identifying retinal breaks. In our study, the overall rate of intra- or post-operative retinal breaks was 13.6%. The incidence of intra- or post-operative breaks was significantly lower in patients treated for ERM (8.5%), or those who underwent DV/vitrectomy for floaters (5.7%) compared to those who underwent vitrectomy for MH or VMT (20.6%, p = 0.001).

A previous cross-sectional study examined the incidence of vitreopapillary adhesion, or anomalous PVD that involves persistent adherence of vitreous to the optic disc, in patients with full thickness MH, lamellar holes, dry age-related macular degeneration, and macular pucker, and found that by far vitreopapillary adhesion was most prevalent in patients with MH compared to the other pathologies described [23]. This study suggests that while anomalous PVD may be the initial event in pathogenesis of MH or VMT, presence of vitreopapillary adhesion may alter the subsequent disease course and traction vectors, leading to development of MH or VMT. Our study builds on this evidence, suggesting that vitreoretinal adhesion may play a similar role in the pathogenesis of these conditions. These patients, therefore, may be predisposed to peripheral peri-operative retinal breaks during surgery for the same underlying reasons that their MH or VMT initially developed, by increasing vitreous adhesion to the retina.

In our study, those who underwent 23-gauge PPV had a much higher rate of intra-operative breaks (19.4%) versus those who underwent 25-gauge vitrectomy (3.8%), and gauge was an independent predictor of the primary outcome on multivariable analysis. However, two retrospective studies investigating retinal breaks after 23-gauge and 25-gauge vitrectomy, one for MH (47 patients) and one for proliferative diabetic retinopathy (85 patients), found no significant difference in outcomes [24, 25]. A prospective study comprising 184 patients with and without diabetic retinopathy demonstrated no difference in outcomes or complications for 23-gauge vs. 25-gauge sutureless microincision vitrectomy [26].

Compared to the 25-gauge systems, 23-gauge vitrectomy uses larger bores and thus higher fluidics, creating higher linear velocity flow, and may use a lower cut rate. Additionally, the peripheral vitreous may have been more aggressively removed in cases using 23-gauge vitrectomy, as it is more difficult to rotate the eyes to reach the periphery with the more flexible 25-gauge probe. It is also possible that surgeons more often elected to use the 23-gauge vitrector in challenging cases which were more likely to have intra-operative retinal breaks noted, which we cannot account for in our limited retrospective study design. For example, the use of 23-gauge vitrectomy was much higher in patients with MH/VMT (37.5%) compared to patients with ERM (24.1%) and DV/vitreous opacities (22.9%). Most surgeries (70.1%) employed 25-gauge vitrectomy, thus offering a smaller sample size of 23-gauge vitrectomy cases. Treatment modalities also differed between patients with MH/VMT and patients with ERM, notably the use of gas, endolaser or cryotherapy, which was increased in patients with indication of MH/VMT, as would be expected based on accepted treatment modalities.

The major limitations of the study include its observational design and its retrospective collection of patient data from a single health system. However, the Kellogg Eye Center has 11 retinal surgeons who vary in training and experience, each of whose cases were included in the study. Additionally, the operative report and patient charts are often not clear and consistent regarding how often surgeons were required to create a PVD, which may be an important consideration in determining risk of peri-operative breaks. The analyses did not account for surgeon experience, fellow or resident participation in surgery, or advances in surgical technology over the 13-year span of the study. Our rate of tears is higher than might be expected in the overall population and especially in the 23-gauge subgroup. We arrived at this number conservatively, given the fact that many practitioners do not directly report intraoperative tears in their operative reports, and the numbers were gleaned from analysis of subsequent patient chart review. It is possible that this analysis overestimates the number of tears present in the patient population, and we recognize this as a limitation of the study, albeit unavoidable given its retrospective nature.

In conclusion, the presence of MH and VMT, as well as use of 23-gauge vitrectomy may be significant risk factors for the development of peri-operative retinal breaks during PPV. This knowledge is important for clinicians in counseling patients on risks and benefits of surgery, as well as in risk-stratification for those in whom PPV is being considered. In addition, this information may spur clinicians to exercise heightened caution when performing a thorough peripheral exam to evaluate for retinal breaks. Further prospective studies are needed to better characterize the risk of peri-operative breaks based on these indications and technical considerations.
